# A Delphi-Based Validation of Consumer Food Labelling Lessons for South African Consumers

**DOI:** 10.1177/10901981261436046

**Published:** 2026-04-12

**Authors:** Shakun Naicker, Ashika Naicker, Heleen Grobbelaar

**Affiliations:** 1 Durban University of Technology, Durban, South Africa

**Keywords:** consumer, food labelling, intervention, Delphi, experts

## Abstract

Food labelling plays a critical role in informing consumers about the nutritional quality, safety, and composition of food products. However, many South African consumers struggle to understand or effectively use food labels. This gap in health literacy highlights the need for culturally appropriate educational interventions. In response, consumer food labelling lessons were developed using evidence-based literature and findings from a South African consumer food labelling survey. In this qualitative study, the Delphi-based validation approach was employed to ensure relevance, clarity and appropriateness of the lessons. A panel of seven South African experts from academia, health care, and the food industry participated in two rounds of feedback. Consensus was reached in Round 2, and a final confirmation process was conducted in Round 3. Key themes that emerged in Round 1included the need to simplify language and reduce scientific jargon. At least 80% agreement was reached on the need for more visual examples and simpler explanations. By Round 2, ≥85% agreement was achieved that these revisions had been incorporated effectively. In Round 3, the reviewers confirmed consensus on the lessons’ relevance, clarity, and cultural appropriateness, and the research team considered the final conclusions and approved the lessons as suitable for implementation in socioeconomically diverse populations in South Africa. This work adds to the limited South African literature on food label education by presenting a validated, culturally contextualised intervention. It holds public health relevance as a model for improving food label literacy and empowering healthier food choices among South African consumers.

It is a well-known fact that at a global level, the rapidly increasing rate of overweight and obesity calls for bold, stringent, and decisive leadership in nutrition and strategic health plans (Gibson-Moore, 2021). Governments worldwide are urged by the World Health Organization (WHO) to construct comprehensive and effective corrective measures to address these trends (UNICEF, 2021). The burden of overweight, obesity, and noncommunicable diseases (NCDs) has been reported to be increasing annually in low- and middle-income countries (LMICs), including South Africa (SA). When disaggregated by gender (females *n* = 19,982; males *n* = 10,099), recent data from the South African National Food and Nutrition Security Survey (NFNSS) report indicated that 67.9% of females and 38.2% of males either overweight or obese (Simelane et al., 2024), highlighting the continued and increasing prevalence of overweight and obesity in SA. Given the strong association between poor dietary choices and rising obesity rates and NCDs, addressing what people consume and how they are informed about their food consumption becomes crucial. Despite efforts by the South African government and global agencies to promote food label usage, evidence suggests that the current labelling format may not effectively improve public health outcomes. There is limited evidence that South African consumers adequately understand the current food labelling practices or use them to make informed food choices (Bopape et al., 2021; Koen et al., 2018; Mahlangu et al., 2024). In addition, the challenge of language and varying educational levels spread across SA (Statistics South Africa, 2022) is an added factor, causing a barrier to consumer use of food labelling. These gaps are indicative of the results previously obtained in food label studies in SA (Koen et al., 2018; Mahlangu et al., 2024). In the context of global studies, South African consumers are not very different from other consumers around the world in this regard (Cowburn & Stockley, 2005; Kempen et al., 2012; Mackey & Metz, 2009). Food labelling plays a fundamental role in informing consumers about the nutritional quality, safety, and composition of food products, yet consumers underutilise it (Kempen et al., 2012; Moore et al., 2018). Furthermore, researchers have duly emphasised that for nutrition labels to impact public health positively, they depend on the consumer’s ability to decipher and use this information (Moore et al., 2018). This highlights the importance of ensuring that consumers are empowered with clear, accessible, and actionable information about what is in their food. Food labelling, particularly when designed with public health objectives in mind, is one such strategy that links dietary intake to health outcomes (Newman et al., 2018). By improving transparency and enabling informed decision-making, food labels serve as a critical interface between food systems and consumer health (Food and Drug Administration [FDA], 2018). Food labelling is no doubt difficult for consumers to interpret; however, the lack of motivation to make use of it by many consumers is repeatedly raised as a concern in past literature (Mahlangu et al., 2024; Mehanna et al., 2024). Researchers link the high engagement with shelf-life information to consumer perception about their concerns about the quality and safety of the food product (Moore et al., 2018), while, on the contrary, the lack of consumer concern about the importance of nutrition information labelling including serving sizes on a product is concerning since understanding and usage of this information is important in making informed decisions. Front-of-pack nutrition labelling (FOPNL) tools have been at the forefront of many discussions related to health policy and nutrition strategies to curb obesity and NCDs (World Health Organization [WHO], 2019, 2020). The South African Department of Health (DOH) has proposed a FOPNL tool in the draft R3337 food labelling regulation in April 2023 (Department of Health [DOH], 2023) as a measure to strengthen the framework of the national strategic health plan in SA. The draft R3337 is a good example of how the DOH wants to improve public health in the future, but with that said, education is still key to enabling healthy food choices and improving food and health literacy, such as labelling information. While it is anticipated that Draft R3337 will bring valuable contributions, it is important to recognise that sustainable, healthy food choices ultimately depend on education. Strengthening food and health literacy, particularly around interpreting labelling information, is key to empowering consumers to make informed decisions about food product purchasing. Previous nutrition-related educational interventions recorded in literature have highlighted their role in changing behaviour, shifting consumer mind-set and increasing usage of labels when the interventions are well managed and documented (Campos et al., 2011; Moore et al., 2018).

The Delphi method is a structured consensus technique used when empirical evidence is limited or unclear. It facilitates expert group communication to address complex issues and improve decision-making (Humphrey-Murto & De Wit, 2019; Jünger et al., 2017; Turnbull et al., 2018). In this study, the Delphi method was used to adapt and validate the consumer food labelling lessons (CFLLs), ensuring their relevance and cultural appropriateness aligned with the values, beliefs, customs and social norms, ensuring that the content delivered was respectful, relevant and sensitive for SA.

## Method

### Lesson Development

A literature review was conducted to identify existing food labelling educational interventions and materials to promote public awareness, drawing on South African and international sources to establish a strong foundation for the study’s educational content.

Resources from the Food and Drug Administration (FDA), and South African organisations such as the Heart and Stroke Foundation, the Cancer Association of South Africa (CANSA), and Woolworths informed the development of the CFLLs (Cancer Association of South Africa [CANSA], 2025; FDA, 2018; Heart and Stroke Foundation South Africa, 2023; Woolworths, 2025). These materials highlighted key topics like front-of-pack labels, date coding, allergens, and nutritional information. Insights from a South African consumer food labelling study further guided the inclusion of relevant content to address knowledge gaps and enhance public understanding (S. Naicker et al., 2026). The lesson content, objectives, and rationale were constructed using a ‘global best practice’ approach. This was translated into the South African context so that the lessons would be relatable and to enhance theory awareness, understanding, usage, and attitude towards food labels.

### Selection of the Expert Panel

Consideration was given to the criteria for selecting experts (Lemmer, 1998) for the Delphi review. Experts were purposefully selected based on the criteria as identified by Humphrey-Murto and De Wit (2019). The selected experts reflected different dimensions of expertise and were well known for their contribution to this field of work in SA, and included dieticians, academics and published authors with community experience and similar interests in food labelling lessons. Even though there are 11 official languages in SA, 60% of the population speak one of the four major languages being isiZulu, isiXhosa, Afrikaans and English (Statistics South Africa, 2022); therefore, the selected panel represented cultural inclusion of the four major languages spoken in SA. Experts were recruited through email, requesting their participation in the review through informed consent after receiving a letter of information that detailed their role in participating in the Delphi review and what was required of them. Table 1 provides details of the institution or organisation to which experts belong.

**Table 1.** Affiliation of Selected Experts.

Panel expert	Organisation
Expert 1	Traditional University
Expert 2	University of Technology
Expert 3	Grains and Legumes Nutrition Council
Expert 4	Industry Expert – Food Labelling
Expert 5	Traditional University
Expert 6	University of Technology
Expert 7	Healthy Living Alliance (HEALA)Technical Expert

### Delphi Questionnaire Development

The Delphi questionnaire was constructed based on current literature, which suggests that the questions about the lesson content for Delphi Round 1 be qualitative, meaning open-ended and closed-ended Likert-type style, which allowed the experts to be creative, to generate ideas and the opportunity to agree or disagree with the content (Chuenjitwongsa, 2017). The panel needed to examine the CFLLs for appropriateness of language for comprehension, teaching aids like labels (visuals) and activities (interactive content) for participants, to evaluate the content and flow of the material; is it accurate and scientifically sound, to evaluate whether all the elements of food labelling (e.g., nutritional content, ingredients, portion size, and claims) has been included in the CFLLs, and importantly, if the lessons were relevant and met the objectives of what this study intended to achieve. According to Chuenjitwongsa (2017), the response rate of the experts should not fall below 70%, and if the rate falls below 70%, the next round of review cannot be executed. The Delphi Round 2 questionnaire was based on the outcome of recommendations and comments received from the experts in Round 1. In Delphi Round 3, the research team considered the recommendations or comments made by the reviewers in the final review. The number of rounds is variable and dependent upon the purpose of the research, and it is suggested by experts that a two- or three-round Delphi is sufficient for most research (Delbeq et al., 1975).

### Ethics

Ethical approval was also obtained from the Durban University of Technology Institutional Research Ethics Committee (IREC 201/23). The letter of information described the nature of the study, the requirements for participation, and the necessary protocols to ensure anonymity was maintained throughout the study.

### Data Analysis

The Delphi approach encouraged expert creativity, allowed for idea generation, and provided an opportunity for experts to express agreement or disagreement with the lesson content. A consensus was defined as ≥80% of experts rating an Item 4 or 5 on a 5-point Likert-type scale. A median score of 5 indicated strong agreement, while an interquartile range (IQR) of 0 reflected minimal variation in responses.

## Results

### Delphi Round 1

In Round 1, the key themes to simplify language, increase visuals, avoid jargon and reduce the length of some lessons were consistently highlighted by all reviewers. Tables summarising feedback and modifications made in Round 1 are presented in Table 2. Focus was given to the objectives of the lessons due to this being an important component of the food labelling educational intervention. With a 100% response rate, an agreement score of 100% (*M* = 4.857), indicate that the objectives of the educational lessons were accurate and sound. In addition, 71.4% (*M* = 4.000) of the reviewers agreed that the language of the lessons should be simplified and to limit technical and scientific jargon and expressed that the examples used to describe scientific information were good and suggested that more of these examples and visuals be used throughout the lessons. Reviewers (100%; *M* = 4.714) strongly agreed that the lesson content and flow of the material were accurate and scientifically sound.

**Table 2.** Delphi Round 1, Summary of Reviewer Feedback and Modifications Made.

Question	*M*	*SD*	Median Score	IQR	Round 1 % agreement	Summary of expert comments	Modifications made
Please evaluate Lessons 1 to 6 against the lesson objectives for lessons that will be administered to South African adults, and if the objectives are accurate and sound.	4.857	0.378	5	0	100	Well-aligned, clearly stated, appropriate for target audience	Additional visuals added to lessons 1–5; pacing adjusted
Please evaluate whether the language used in the material is suitable for comprehension.	4.000	1.155	4	0	71.4	Language too technical; simplify scientific terms	Language simplified across all lessons
Please evaluate the content and flow of the material; is it accurate and scientifically sound?	4.714	0.488	5	0	100	Scientifically sound but lengthy; add refresher	Refresher included at start of each lesson
Evaluate the appropriateness of the teaching aids like labels (visuals) and activities (interactive content) for participants.	4.714	0.488	5	0	85.7	Effective visuals; ensure image clarity and referencing	Images referenced and improved
In your opinion, are the lessons engaging and informative? What improvements could be made?	4.714	0.488	5	0	85.7	Informative and interactive; more practical examples suggested	Practical activities and examples added
Have all the elements of food labelling (e.g., nutritional content, ingredients, portion size, and claims) been included in the lesson pack, which are most important for consumers to understand?	4.571	0.535	5	0	100	Focus on fat, sugar, salt; too much theory in lessons 5 and 6	Nutrition info simplified; lessons 5 and 6 shortened

A higher level of consensus was observed among the reviewers, with 85.7% (*M* = 4.714) agreeing that the teaching aids, such as labels (visuals) and activities (interactive content), were suitable for the food labelling educational intervention. All reviewers agreed (*M* = 4.571) that elements of food labelling (e.g., nutritional content, ingredients, portion size, and claims) included in the lesson pack were most important for consumers to understand. Some reviewers advised that the nutrition Lessons 5 and 6 should focus on the three nutrients (sugar, fat and salt).

In the first round of review, there was strong agreement among the experts regarding the CFLLs. The median rating was 5, indicating that reviewers strongly agreed that the lessons’ objectives were accurate, the content and flow were appropriate, the teaching aids were acceptable, the lessons were engaging, and the coverage of food label elements was accurate. However, they noted that the language used needed further simplification. The IQR was 0, demonstrating very little variation in responses and suggesting a high level of consensus.

### Modifications

Based on feedback from Round 1, the food labelling educational intervention was modified to include a shorter and simplified version of the lessons, especially Lessons 5 and 6, aligning with reviewer recommendations to reduce lesson content to focus on three nutrients. The language was simplified, and more images and activities were added to aid understanding, and a refresher from the previous lesson was included at the start of each lesson.

### Delphi Round 2 – Refinement and Consensus Building

In Round 2, consensus was achieved (≥85% agreement). The revised CFLLs were well received: the reviewers acknowledged the added visuals, reduced content and simplified text in their final response. The Round 2 questionnaire, together with the revised food labelling educational lessons and the consolidated feedback from Round 1, was sent back to the panel of experts for the second review to be conducted. Round 2 achieved an 85.7% expert response rate, with only one reviewer unable to provide a second review. The Delphi Round 2 survey questionnaire is presented in Table 3. The reviewers were in 100% agreement that after the second round of reviews, the objectives of the food labelling lessons were met (100%; *M* = 4.833). Reviewers (100%; *M* = 4.833) agreed that the lessons were presented in relatable and simplified language to enable the participants to grasp concepts more easily. A refresher (a quick recap of the previous lesson content) was included at the start of the new lesson to remind participants about the previous lesson. Reviewers were in 100% (*M* = 4.667) agreement that the content was scientifically accurate and easy to understand. It was also agreed 100% (*M* = 4.500) that the training activities and materials, teaching aids (e.g., visuals, interactive content, and activities) were appropriate and effective for enhancing participant engagement. Furthermore, 100% (*M* = 4.833) of the reviewers agreed that the added images gave more clarity and aided in supporting the theory, and the shortened Lessons 5 and 6 were well received, and 100% (*M* = 4.667) of the reviewers agreed that the lessons would encourage participation. Most importantly, there was 100% (*M* = 4.833) agreement that the lessons sufficiently covered the most important elements of food labelling (e.g., nutrition content, ingredients, serving size, and claims). All reviewers agreed that the revisions made to the CFFLs addressed the recommendations provided during Round 1. Reviewers reached consensus that the lesson pack now sufficiently covered the most important elements of food labelling (e.g., nutrition content, ingredients, serving size, and claims).

**Table 3.** Delphi Round 2, Summary of Reviewer Feedback and Modifications Made.

Question	*M*	*SD*	Median	IQR	Round 2 % agreement	Summary of expert comments	Modifications made
Have the revisions made to the food labelling educational intervention addressed the recommendations provided during Round 1?	4.833	0.408	5	0	100	Minor wording changes; QUID spelling corrected	The spelling of QUID was corrected, and changes were made to wording as suggested
Are the objectives of the revised food labelling educational intervention accurate, sound, and aligned with the intended outcomes?	4.833	0.408	5	0	100	Clear alignment with intended outcomes	All recommendations added in the revision enhancing the accuracy of the lessons further to achieve its intended outcome
Has the language been simplified enough to ensure clarity and reduce technical or scientific jargon?	4.833	0.408	5	0	100	Improved understanding for target group	Language was simplified through the lesson plan, and technical jargon was reduced
Is the revised content scientifically accurate and easy to understand for the target audience?	4.667	0.516	5	0	100	Appropriate and accessible content	Language was simplified, less use of extracts from draft R3337 regulation, lessons shortened
Are the revised teaching aids (e.g., visuals, interactive content, and activities) appropriate and effective for enhancing participant engagement?	4.500	0.548	4.5	0	100	Visuals enhanced; interactive group work added	Additional visuals were added to aid understanding with more activities to engage consumer participation
Do you feel the balance of text and visuals is now appropriate?	4.833	0.408	5	0	100	More balanced presentation noted	All lessons included appropriate visuals, more visuals used to enable teaching
Does the lesson pack now sufficiently cover the most important elements of food labelling (e.g., nutrition content, ingredients, serving size, and claims)?	4.833	0.408	5	0	100	Critical components included; jargon removed	Lesson 5 and 6 significantly shortened, and lessons covered important food labelling topics

In the second round of review, there was continued strong agreement among the experts, with a median rating of 5. This indicated that the revisions made to the CFLLs effectively addressed the recommendations provided during the first round. The IQR remained at 0, reflecting minimal variation in responses. This suggests a high level of consensus among the reviewers, with most assigning the highest possible rating to the revised lessons.

### Modifications

Based on the 100% agreement and consensus achieved in Round 2 following the modifications made to the lessons post Round 1, there was only one additional modification to the lesson pack, which was to include educational material about vegan and vegetarian claims and what they meant.

### Delphi Round 3: Finalisation

The research team finalised the CFLLs with final reviewer verification comments presented in Table 4, following a structured process guided by literature (Hasson et al., 2008; V. N. Naicker et al., 2024). In Round 3, the research team checked the responses from Round 2 for agreement. The reviewers confirmed consensus, and their recommendations were implemented, as advocated by Hasson et al. (2008). According to the literature, the final Delphi round is confirmatory (Hasson et al., 2008) and serves to validate near-final content (Lemmer, 1998). Since consensus had been achieved earlier, this last round was a final sign-off (Murphy et al., 1998; Powell, 2003). The research team gave final confirmation and sign-off for the intervention, approving its suitability for implementation (Mead & Mosely, 2001).

**Table 4.** Final Reviewer Verification Comments.

Topic	Reviewer consensus	Summary feedback
Lesson 1: FOPNL moved to earlier lesson	All agreed	Improved structure and sequencing of content
Pictorial aids in Lesson 2	All agreed	Enhances clarity and comprehension
Activities in Lessons 3 and 4	All agreed	Supports learning of complex topics like QUID
Lesson 5 focus: fat, salt, sugar	All agreed	Aligned with goal of simplicity and practicality
Lesson 6: Claims simplified	5 Yes, 1 Suggestion	Consider adding vegan/vegetarian claim info in future

## Discussion

### Overview

This study used the Delphi method to validate a series of CFLLs designed for South African adults. The Delphi process, comprising two structured rounds and a final verification stage, facilitated expert consensus on lesson accuracy and clarity. Cultural appropriateness was evaluated according to the selection of well-known South African food brands used in the lesson content, and whether the images, colours and symbols used were culturally sensitive and free from negative connotations. Another aspect that was considered by the panel was whether the content was inclusive and not bias towards one cultural group. The high agreement levels achieved across key domains, including lesson objectives, language simplicity, scientific accuracy, visual supports, and overall relevance, emphasise the effectiveness of this consensus-building approach.

### The Effectiveness of the Delphi Method in Refining Educational Tools

The Delphi method is particularly effective as a research tool in situations where knowledge about a problem or phenomenon is limited (Skulmoski et al., 2007; Whittal et al., 2021). The Delphi method is highly valuable for enhancing understanding of complex issues, identifying opportunities, evaluating potential solutions, or generating forecasts (Skulmoski et al., 2007; Whittal et al., 2021). Its structure, which typically includes at least two review rounds, allows experts to brainstorm in the first round and focuses subsequent rounds on refining and reaching consensus (Schmidt, 1997). Ongoing verification throughout each Delphi round is critical for ensuring reliability and should be embedded within the research design (Adler & Ziglio, 1996; Delbeq et al., 1975; Linstone & Turoff, 1975), as implemented in this study. The third round of review further confirms completeness and finalises the CFLLs, allowing the project to move confidently into implementation (Schmidt, 1997).

### The Importance of Tailoring Health Education to Literacy Levels and Cultural Context

The iterative nature of the Delphi method ensured that the feedback received in the initial round informed tangible improvements to the lesson content, including simplified language, the addition of engaging visuals, and reduced technical jargon. These changes directly addressed common barriers to label comprehension identified in the South African context, such as language diversity, low health literacy, and limited nutrition knowledge (Koen et al., 2018; Mabotja et al., 2021). In SA, there are significant challenges associated with language, literacy and legibility of printing on labels exist, resulting in a lack of food label awareness, interpretation, and usage (Todd et al., 2021). Several gaps and challenges in food labelling education were identified in past literature studies that have resulted in barriers to success in achieving food labelling awareness, understanding, interpretation and usage across the globe and, in particular, SA (Gibson-Moore, 2021; Koen et al., 2018). The barriers to food label comprehension are especially prevalent among those with low general literacy and those with limited health literacy skills (Mabotja et al., 2021). To help improve understanding of the theory, more visuals were added to the lessons to enhance understanding of the theory, as visuals simplify interpretation (Alabi, 2024).

### Comparison with Similar Interventions in Other Countries

Globally, structured food labelling education has been shown to drive significant changes in consumer knowledge and behaviour. In India, for example, an intervention with 200 adolescent students (13–15 years) using charts, models, posters and interactive lessons demonstrated substantial improvements in understanding and interpreting food labels (Sindhu & Madaiah, 2023). Pre- and postintervention questionnaires revealed marked increases in label comprehension and usage, including knowledge of best-before dates (Sindhu & Madaiah, 2023). Similarly, a South Korean study using pre- and postquestionnaires with the same group demonstrated the effectiveness of targeted education. The study that measured changes in knowledge and attitudes on consumer awareness of food additives found that 33.1% of consumers believed additives were unsafe and expressed discomfort (Lee et al., 2013). Postintervention, 78% considered them safe. In Soweto, South Africa, participants initially struggled to understand a warning label (Bopape et al., 2021). Following a nutritional education intervention, they immediately grasped its meaning and expressed positive attitudes towards the label (Bopape et al., 2021). In 2016, Chile introduced front-of-package warning labels showed a positive response across all demographics, with a decline in purchases of products high in sugar, fat, and sodium, reflecting improved public perceptions and attitudes towards informed food choices (Taillie et al., 2020). Therefore, evidence of nutrition label education interventions globally has successfully demonstrated improved consumers’ awareness and knowledge of food labels, and food label usage and these interventions will impact future dietary behaviour by prompting healthier food purchases (Chin et al., 2023). These findings align with other theory-driven studies showing that well-designed workbooks, regular lessons, and community engagement significantly improve nutrition literacy and confidence (Cannoosamy et al., 2014; Dutta & Patel, 2017; Moitra et al., 2021).

A systematic review of 17 studies confirmed that education significantly improves understanding and use of nutrition labels (Moore et al., 2018), with researchers concluding that general nutrition knowledge and literacy are vital for promoting healthier dietary decisions. In SA, the ongoing challenge of food insecurity (Statistics South Africa, 2022) means many people prioritise quantity over nutrition. It is vital to pursue policies that enable widespread understanding and use of labels. Education that optimises comprehension and use of nutrition labels may improve the impact of this information on dietary health. Researchers also explain that demographic determinants are important, because the incidence of unhealthy eating habits is known to be unequally distributed across social classes (Campos et al., 2011). Lower use of nutrition information is seen among individuals from lower socioeconomic backgrounds, due to lower nutrition knowledge, lower interest in healthy eating, or other factors such as cost of food, accessibility to healthy food, level of education and age. Time, resources and commitment must be combined to focus on innovative technological applications to achieve widespread learning capability that will be accessible, uncomplicated, and available in SA’s official 11 languages to all South Africans. These findings support earlier theory-driven research in nutrition education in that workbooks, well-structured lessons, weekly meetings, and community-based lessons improve confidence in and enhance perceived advantages of nutrition education (Cannoosamy et al., 2014; Dutta & Patel, 2017; Moitra et al., 2021).

With the draft R3337 labelling legislation still under review, an opportunity exists to embed an educational component within it, ensuring consumer literacy in SA improves alongside new labelling requirements. Such measures could support equitable access to nutrition information, especially for lower socioeconomic groups, making labels an effective tool for promoting healthier choices and alleviating strain on the public health system. The Delphi method, with its structured and flexible approach, provides a valuable framework for such research, ensuring rigour and consensus across expert inputs, ultimately supporting a deeper understanding of nutrition education and its role in public health policy. The findings highlight the urgency for policymakers to mandate clearer labelling standards and support these with educational campaigns to enable South Africans to use FOPNL effectively. Without consumer education, the initiative may fail to achieve its intended public health benefits. In a context where many South Africans face food insecurity (Statistics South Africa, 2025), making healthy choices is challenging due to rising food prices and limited access. Even with tax-exempt staples like brown bread and maize, nutrition and affordability remain pressing issues. More needs to be done to incentivise healthier purchasing decisions, especially for economically disadvantaged groups.

### The Potential Role of These Lessons in National Health Promotion Strategies

In response to rising rates of NCDs such as obesity, hypertension, and diabetes, food labelling policy has evolved to place a stronger emphasis on FOPNL.

FOPNL tools have been central to global health policy efforts aimed at reducing obesity and NCDs (WHO, 2019, 2020). Front-of-pack nutrition labelling has been established in SA as a cornerstone of its health policy plan, but it may be insufficient without consumer labelling education to drive the healthy food agenda. The draft R3337 exemplifies efforts of the SA DOH to improve public health, but with that said, education is still key to enable healthy food choices and to improve food and health literacy, such as labelling information. While it is anticipated that Draft R3337 will bring valuable contributions, it is important to recognise that sustainable healthy food choices ultimately depend on education. Strengthening food and health literacy, particularly around interpreting labelling information, is key to empowering consumers to make informed decisions about food product purchasing. Given the current policy momentum surrounding FOPNL in SA’s Draft R3337 regulations, these CFLLs are both timely and necessary, given that it is the first culturally tailored and validated curriculum food labelling education in SA, thus supporting current regulatory developments in food labelling. The lessons could be potentially implemented through strategies in corporate wellness programmes, community health forums and digital platforms making it accessible for all South Africans. Legislation may mandate changes to labels, but consumer education will ensure they are effectively utilised. Thus, CFLLs can complement regulatory reforms by enhancing consumer agency and supporting healthier food choices.

### Strengths and Limitations

A key strength of this study lies in its use of the Delphi method, which enabled a rigorous, structured consensus-building process involving a multidisciplinary panel of South African experts in food science, nutrition, and public health. The two iterative rounds of review and a final verification stage allowed for refinement and validation of the CFLLs, ensuring their relevance, clarity, scientific accuracy, and cultural appropriateness. The engagement of experts from diverse yet relevant fields enhanced the credibility and robustness of the feedback received, resulting in well-aligned, learner-centred educational content tailored to the South African context. In addition, the high response rate across the Delphi rounds and the unanimous agreement achieved in the final round further highlight the methodological strength and the reliability of the content developed. However, the study is not without limitations. The scope of this article is limited to the expert validation phase of the study and excludes the subsequent consumer testing phase. The expert panel was limited to seven participants, all based in SA. While this ensured contextual relevance, it may limit the generalisability of findings to other low- and middle-income country settings with different linguistic, cultural, or regulatory contexts. The content developed was limited to English, and the other languages were not considered in the scope of this study.

## Conclusion

The finalised CFLLs reflect an evidence-based, accessible, and learner-centred educational package that aligns with the objectives of promoting public health and food literacy. The validated food labelling lessons are appropriate for South African consumers and can be used in health education, schools, and community settings. Subsequent evaluations should focus on changes in consumer behaviour, knowledge retention, and the scalability of CFLLs across provinces and language groups. Partnerships with government departments, civil society, and the food industry will be key in broadening the reach and impact of this intervention. Education interventions are paramount to improve consumer food labelling literacy. When developing educational lessons, they must be solidified, and the Delphi method should be applied to guide their purpose in targeting nutrition education.
